# 

**Published:** 1860-12

**Authors:** 


Published Monthly, at $3 per Annum in Advance.

### ATLANTA

### MEDICAL AND SURGICAL

### JOURNAL.

J. G. WESTMORELAND, M. D.,

Professor of Materia Medica and Medical Jurisprudence in the Atlanta Medical College,

EDITOR AND PROPRIETOR.

*Pax et scientia, sed veritas sine timore.*

Vol. VI. DECEMBER, 1860. No. IV.

ATLANTA, GA:

ATLANTA INTELLIGENCER PRINT.

1860.

Each Number contains Sixty-Four Pages.

